# Zygomaticus activation through facial neuromuscular electrical stimulation (fNMES) induces happiness perception in ambiguous facial expressions and affects neural correlates of face processing

**DOI:** 10.1093/scan/nsae013

**Published:** 2024-02-08

**Authors:** Themis Nikolas Efthimiou, Joshua Baker, Alasdair Clarke, Arthur Elsenaar, Marc Mehu, Sebastian Korb

**Affiliations:** Department of Psychology, University of Essex, Colchester CO4 3SQ, United Kingdom; Department of Psychology, University of Essex, Colchester CO4 3SQ, United Kingdom; Department of Psychology, University of Essex, Colchester CO4 3SQ, United Kingdom; ArtScience Interfaculty, Royal Academy of Art, Royal Conservatory, The Hague 2514 AN, Netherlands; Department of Psychology, Webster Vienna Private University, Vienna 1020, Austria; Department of Psychology, University of Essex, Colchester CO4 3SQ, United Kingdom; Department of Cognition, Emotion, and Methods in Psychology, University of Vienna, Vienna 1010, Austria

**Keywords:** fNMES, facial feedback, face perception, event-related potentials, embodiment

## Abstract

The role of facial feedback in facial emotion recognition remains controversial, partly due to limitations of the existing methods to manipulate the activation of facial muscles, such as voluntary posing of facial expressions or holding a pen in the mouth. These procedures are indeed limited in their control over which muscles are (de)activated when and to what degree. To overcome these limitations and investigate in a more controlled way if facial emotion recognition is modulated by one’s facial muscle activity, we used computer-controlled facial neuromuscular electrical stimulation (fNMES). In a pre-registered EEG experiment, ambiguous facial expressions were categorised as happy or sad by 47 participants. In half of the trials, weak smiling was induced through fNMES delivered to the bilateral Zygomaticus Major muscle for 500 ms. The likelihood of categorising ambiguous facial expressions as happy was significantly increased with fNMES, as shown with frequentist and Bayesian linear mixed models. Further, fNMES resulted in a reduction of P1, N170 and LPP amplitudes. These findings suggest that fNMES-induced facial feedback can bias facial emotion recognition and modulate the neural correlates of face processing. We conclude that fNMES has potential as a tool for studying the effects of facial feedback.

## Introduction

Embodied cognition theories suggest that the recognition of facial expressions is facilitated by facial mimicry—i.e. the spontaneous imitation of perceived emotional faces—and the accompanying changes in facial feedback (Niedenthal, 2007; Wood *et al*., 2016a). This has been supported by studies that activated or blocked facial muscles. For example, simulating a smile by holding a pen between the teeth can make people perceive happy faces and bodies faster (Marmolejo-Ramos *et al*., 2020) and improve working memory for ambiguous happy faces (Kuehne *et al*., 2021). Conversely, interfering with facial feedback by applying a hardening gel to the face reduces the accuracy in matching emotional facial expressions (Wood *et al*., 2016b), and paralysing facial muscles with Botox injections makes slightly emotional facial expressions appear less emotional, and slows down their recognition (Baumeister *et al*., 2016).

Facial manipulation techniques can also influence the visual processing of emotional stimuli, as measured by event-related potentials (ERPs). When watching emotional faces, a larger P1 has been reported in trials with more facial mimicry (Achaibou *et al*., 2008) and a smaller P1 was found in individuals with high alexithymia traits when facial feedback was altered using restrictive facial gel masks (Schiano Lomoriello *et al*., 2021). Another component relevant to facial recognition is the N170 (Eimer, 2011). Sel *et al*. (2015) found that when participants simulated a smile, by holding a pen between the teeth, N170 amplitude to neutral facial expressions was increased, suggesting that the facial feedback manipulation affected early visual face processing. In contrast, Achaibou *et al*. (2008) reported reduced N170 amplitudes in trials with greater facial mimicry, and Schiano Lomoriello *et al*. (2021) found no significant N170 effects when modulating facial feedback through a hardening face gel. Holding a pen between the teeth may also enhance the N400 component to faces (Davis *et al*., 2017). Finally, the late positive potential (LPP) is modulated by the emotional content, ambiguity and intensity of faces (Liu *et al*., 2012; Calvo *et al*., 2013), but it remains unknown if it is also sensitive to facial feedback effects.

While the facial manipulation methods listed above have played a crucial role in investigating facial feedback effects, they suffer from certain limitations. For instance, Botox injection is an invasive procedure that is primarily administered to female participants, and its effects last several months. It is therefore not the ideal choice for non-invasively testing participants of both genders and capturing rapid effects of facial feedback manipulations. Further, participants often encounter difficulties in adhering to the experimenter’s instructions (e.g. posing the exact intended facial expression), and compliance rates vary depending on the specific facial manipulation method employed (Coles *et al*., 2022). Lastly, modulating facial feedback at specific points in time and for set durations is either impossible or very difficult to achieve using the pen-in-mouth and other techniques discussed above.

Facial neuromuscular electrical stimulation (fNMES) generates controlled facial muscle activations and may thus help to overcome the limitations of the techniques used so far (Efthimiou *et al*., 2022, 2023). Indeed, fNMES allows researchers to selectively target specific muscles—while controlling for the time of onset, duration and intensity of their activation—to generate movements associated with emotional facial expressions. fNMES to the Zygomaticus Major (ZM) and Orbicularis Oculi (OO) muscles was found to increase positive mood and reduce symptoms of depression (Zariffa *et al*., 2014; Kapadia *et al*., 2019; Warren, 2021). However, whether fNMES-induced facial feedback can modulate an individual’s perception of facial stimuli remains to be investigated. Therefore, in this pre-registered study (osf.io/vbnyx), we set out to investigate whether the induction of a weak, short (500 ms) smile through fNMES can influence facial emotion recognition and modulate early visual face processing.

## Methods

### Participants

We recruited 47 mostly right-handed (4 left-handed and 3 ambidextrous) participants (23 females) aged 18–38 years (*M*_age_ = 24.49, s.d._age_ = 5.03). All participants reported having good visual acuity, not having a history or making current use of illicit and/or psychotropic drugs, being free of major heart conditions (e.g. pacemaker), and not having any current or past neurological or psychiatric disorders. An a priori power analysis based on a pilot study and data simulations with the package ‘SimR’ (Green *et al*., 2016) indicated that 40 participants are required to detect a main effect (ß = 0.08) of fNMES on emotion categorisation with 88% power [95% CI, (79.98–93.64)]. For the EEG analysis, nine participants (five females) were excluded due to low-quality data, bringing the final sample size to 38 (*M*_age_ = 24.7 years, s.d._age_ = 4.88). The study was approved by the local ethics committee (ETH1920-0847), and all participants provided written informed consent.

### Materials

**Fig. 1.**
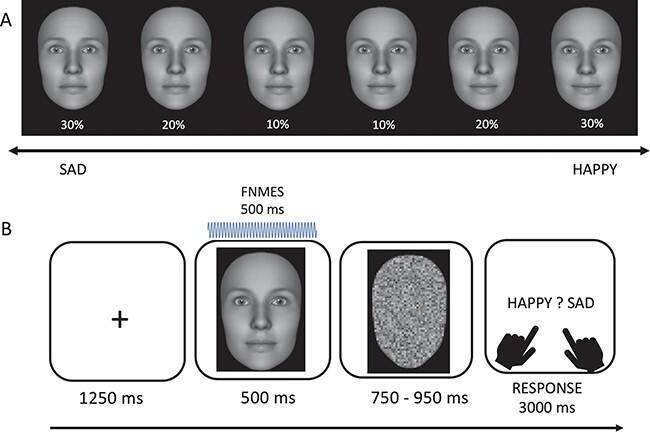
(A) Example of the stimuli used: here is a female avatar, with emotional expressions changing from 30% sadness to 30% happiness in steps of 10%. (B) In each trial, participants viewed a fixation cross for 1250 ms, followed by an avatar face for 500 ms. In the fNMES on condition, electrical stimulation was delivered to the ZM muscle to induce a weak smile. In the off condition, there was no electrical stimulation and participants maintained a neutral expression. Thereafter, participants viewed a scrambled face for a jittered time interval of 750–950 ms, and finally, participants had up to 3000 ms to respond via button press to indicate the perceived emotion of the non-scrambled facial expression (happy or sad).

The stimulus set consisted of 20 avatar faces, 10 males and 10 females, on a black background. Faces were generated with the FaceGen software (www.facegen.com), and their emotional expressions were created based on facial action coding system (FACS) (Ekman *et al*., 2002) using the FACSGen software (Roesch *et al*., 2011; Krumhuber *et al*., 2012). The expressions of happiness included action units (AUs) 6, 7 and 12, while sadness included AUs 1, 4, 7, 11 and 15. Highly ambiguous to somewhat ambiguous expressions with 10%, 20% or 30% happiness and sadness were shown (see Figure 1A), resulting in a total of 120 face stimuli, plus two additional avatars for practice trials. All face images were converted to greyscale and equalised in luminance using the SHINE toolbox in MATLAB (Willenbockel *et al*., 2010).

The Empathy Quotient (EQ; Lawrence *et al*., 2004), Autism-Spectrum Quotient (AQ; Baron-Cohen *et al*., 2006), Multidimensional Assessment of Interoceptive Awareness (MAIA; Mehling *et al*., 2018) and the PANAS questionnaire (Watson and Clark, 1994) were also measured.

### Equipment and fNMES parameters

The delivery of fNMES to the bilateral ZM muscle was achieved using two constant-current electrical stimulators (Digitimer, DS5 https://tinyurl.com/yta3wa3a). A 500-ms long train of biphasic square pulses (100-µs biphasic pulse width and 14-ms delay between biphasic pulses) was delivered at 70 Hz using disposable Ag/AgCl electrodes measuring 16 × 19 mm (Ambu BlueSensor BRS). Stimulation intensity was below 2 RMS mA/cm^2^ following safety guidelines (Efthimiou *et al*., 2023). EEG data were sampled at 512 Hz using an eego sports amplifier (ANT Neuro, https://tinyurl.com/yvnjy5sm) and a 64-electrode waveguard cap—electrodes AFz and CPz served as ground and reference, respectively.

### Procedure

The study consisted of a single session lasting approximately 2 hours, for which participants were compensated with a £25 voucher. Before the laboratory appointment, participants completed a survey administered via Qualtrics, where they were screened for exclusion criteria, were provided basic demographic information and filled out the MAIA, ASQ and EQ questionnaires. The laboratory task was programmed in PsychoPy 3 (v3.2.4; Peirce *et al*., 2019).

Upon arrival, participants were seated in a sound-attenuated booth and were positioned 60 cm from the centre of a 24.5-inch screen with a resolution of 1920 × 1080 and a refresh rate of 360 Hz. The experimenter cleaned the skin of the participants’ cheeks using 70% isopropyl alcohol wipes. Two pairs of disposable electrodes were placed over the bilateral ZM muscles, following electromyography (EMG) guidelines (Fridlund and Cacioppo, 1986). To identify the best positioning of the electrodes and ensure that a weak smile could be induced comfortably, fNMES intensity was gradually increased until visible muscle contractions were observed. On average, fNMES was delivered at 22.60 mA (s.d. = 3.62, range: 14.25–33.75). After the fNMES electrodes and calibration were set up, the EEG cap was gowned and the task began.

Following the completion of the PANAS questionnaire, the participants were provided with written instructions outlining the primary task, which included eight practice trials and a total of 650 experimental trials in pseudorandom order (maximum eight repetitions of the same fNMES conditions and four repetitions of the same facial expression). The main task included 300 trials without fNMES and 300 with fNMES starting at face onset and lasting for 500 ms. In 50 additional ‘fNMES only’ trials, stimulation was delivered without showing a face. The elements and timings for each trial can be seen in Figure 1B. Overall, the experiment lasted 50 minutes, including eight breaks with participants receiving feedback about their accuracy. During the calibration and task, the participants were not able to see their faces, which were however filmed with a webcam.

After the main task, participants rated how (un)comfortable the fNMES had felt. As a reminder, they received another 500 ms of stimulation (without visual stimuli) and rated discomfort on a 100-point visual analogue scale with the anchors 0—‘extremely comfortable’ to 100—‘extremely uncomfortable’; finally, the PANAS was administered for a second time.

### Data preparation and analyses

We followed our pre-registered pre-processing and analysis steps but also included additional exploratory analyses. Two participants who failed two out of the three test items were deemed inattentive and excluded from the analysis. The MAIA, ASQ and EQ were standardised using *z*-score transformation. All participants were retained for the emotion categorisation data, which was cleaned by removing trials with no response (i.e. did not respond within 3000 ms; 0.30% of all trials), and those with a reaction time < 100 ms or >3 s.d. above the mean (4.67% of all trials).

Statistical analyses were conducted in R (R Core Team, 2020), implementing mixed models with the lme4 (Bates *et al*., 2015) and lmerTest (Kuznetsova *et al*., 2017) packages. The first of our pre-registered models tested that fNMES would increase participants’ choices of happiness. It included the fixed effects of emotion (six levels, entered as a continuous variable: the 30, 20 and 10% intensity levels of sadness were coded as −3, −2 and −1, while the 10, 20 and 30% intensity levels of happiness were coded as 1, 2 and 3) and fNMES (on and off; entered as a categorical predictor). The interaction was removed from the random effect’s structure due to singular fits (see Supplemental Material A), and the model formula was: Choice ∼ Emotion * fNMES + (Emotion + fNMES | Participant). A follow-up analysis included several covariates to control for individual differences. A second pre-registered model tested if fNMES influences emotion choice mostly when stimulus emotion is ambiguous. The 30, 20 and 10% emotion intensities were coded as 0, 0.5 and 1 ambiguity, respectively. The formula of the model was: Choice ∼ emotion + fNMES + Ambiguity + Emotion:fNMES + Emotion:Ambiguity + Ambiguity:fNMES + (1 | Participant).

An exploratory analysis examined whether fNMES-induced smiling altered participants’ mood as measured by the PANAS pre- and post-task, using two separate linear regression models, one for positive affect and the second for negative affect.

**Fig. 2.**
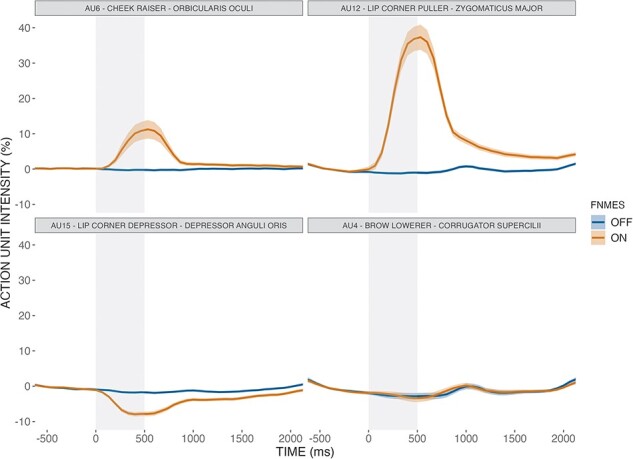
Baseline-corrected results from the OpenFace analysis of video recordings (500–2000 ms) of participants’ faces, based on the FACS (Ekman *et al*., 2002). The activation of four AUs (AU6, AU12, AU15 and AU4), averaged across all trials where algorithm confidence was > 95%, is shown for trials with (orange) and without (blue) fNMES. Notice how fNMES delivery (period indicated by the shaded area) resulted in a 40% activation of AU12, which corresponds to the ZM muscle, followed by a faint activation of AU6 and a relaxation of AU15 (an antagonistic muscle). Importantly, fNMES delivery did not result in AU4 activation (reflecting frowning), which would have been suggestive of a pain response or negative emotion induction. The shaded grey region on the line represents the SE.

The degree of fNMES-induced smiling was captured with video recordings (cut from 500 ms before to 2000 ms after fNMES onset) and estimated with FACS implemented in OpenFace (Baltrusaitis *et al*., 2018). The activation of AUs 6, 12, 4 and 15 during facial stimulation was thus obtained on a scale from 0 to 5 and baseline corrected using the first 1000 ms before face onset. These data were then averaged over the 1000-ms period to capture both the ramp-up and the downtime (see Figure 2).

#### EEG processing and analyses

The EEG data were analysed in MATLAB using the EEGLAB toolbox (Delorme and Makeig, 2004). We followed a previously established procedure for the cleaning of fNMES-induced artefacts (Baker *et al*., 2023). All 650 trials were filtered with a 0.5 Hz high pass and 80 Hz low pass, channels with excessive noise or artefacts were identified through visual inspection and interpolated, line noise was removed using Zapline and Cleanline, and data were epoched from 500 ms before to 800 ms after stimulus onset. We performed independent component analysis (ICA) on the data using the runica function in EEGLAB and removed components representing blinks and fNMES artefacts (see Baker *et al*., 2023 for a detailed description of this approach). Trials were labelled for rejection if values in the pre-stimulus baseline for any channel exceeded ±100 µV. This was performed following the initial channel rejection step, and in all labelled cases, large slow-fluctuating oscillations were observed across all channels. The baseline period was chosen to not include the large amplitudes observed during fNMES. The data were finally filtered with a 40-Hz low pass filter and re-referenced to the common average.

Following pre-processing, we extracted average amplitudes for the following ERP components: P1 (averaged over O1/O2, 80–140 ms), N170 (averaged over P7, TP7, P8 and TP8, 130–190 ms) and LPP (averaged over CPz, Pz and POz, 450–650 ms). To identify the electrodes and times to derive component mean amplitudes, the standard deviation of all channels (mean of all trials from all participants) was plotted over time. Peak deviations were identified through visual inspection. Scalp topography at the timings of identified peaks allowed for the selection of electrode clusters. Finally, we extracted the same ERP component amplitudes from the fNMES-only trials and subtracted them from the fNMES-on trials, therefore removing somatosensory activations associated with receiving stimulation and avoiding contamination of visual evoked potentials (Sel *et al*., 2014; for a similar analysis, see Galvez-Pol *et al*., 2020).

Linear mixed models were fitted for each ERP component including the fixed effects of Emotion and fNMES (formula: Amplitude ∼ Emotion * fNMES + (1 | Participant)). Contrasts were set to sum, and outputs were reported as type 3 ANOVAs. Post hoc comparisons were carried out with emmeans (Lenth, 2023).

## Results

Ratings of discomfort provided to a single 500-ms period of fNMES at the end of the task were generally low (*M* = 30.36, s.d. = 18.71, range: 1.17–70.73). We began by checking whether fNMES-induced smiling altered mood, as measured with PANAS. Both linear regressions revealed no differences between pre-and post-task for positive [β = −0.98, 95% CI (−2.29, 0.34), *t*(82) = 1.47, *p* = 0.145] and negative affects [β = −2.38, 95% CI (−5.58, 0.81), *t*(82) = 1.48, *p* = 0.142]. Overall, there were no changes in positive and negative affects at the start and end of the session.

### Emotion categorisation data

**Fig. 3.**
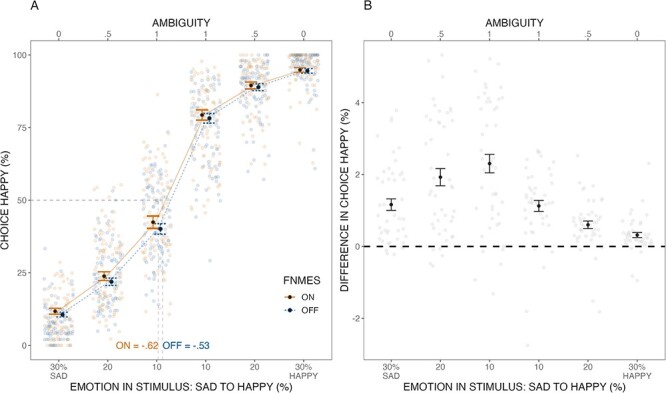
The predicted values for the main effect of fNMES on happy responses to facial stimuli varying from 30% sad to 30% happy. Panel (A) shows the percentage of happy responses across Emotion and fNMES, using the marginal means of the model. Individual dots display participants (jittered to improve visibility), and the dark point reflects the mean with SE bars (SE). Panel (B) displays the mean difference (and SE) in the percentage of happy responses between fNMES conditions (on minus off) across emotion levels. The shaded points represent participant means.

Our first pre-registered analysis was to predict choice by emotion and fNMES (conditional *R*^2^ = 0.59, marginal *R*^2^ = 0.51). A significant main effect of Emotion [β = 0.93, *z* = 28.62, 95% CI (0.86, 0.99), standard error (SE) = 0.03, *p* < 0.001] indicated that participants followed instructions and were overall able to accurately recognise emotional facial expressions. Importantly, a significant main effect of fNMES was found [β = 0.09, *z *= 2.46, 95% CI (0.02, 0.17), SE = 0.03, *p* = 0.014], indicating, as predicted, that more faces were categorised as happy when fNMES was delivered to the smiling muscles (Figure 3A). The interaction between Emotion and fNMES was not statistically significant [β = −0.01, *z *= 0.04, 95% CI (0.04, 0.04), SE = 0.02, *p* = 0.538]. Points of subjective equality (PSEs) showed that faces categorised as happy could contain more sadness when fNMES was delivered (on = −0.62 compared to off = −0.53; Figure 3A). A score computed by subtracting the percentage of faces categorised as happy in the fNMES on minus off condition was positive for all emotion levels and was greater for sad and the most ambiguous expressions (the 10% ones), than for 20% and 30% happy faces (see Figure 3B).

To control for a series of interindividual differences, the first model was followed up by exploratory analyses that also included several covariates (MAIA, EQ, ASQ, PANAS and fNMES-induced discomfort), participants’ ratings of discomfort and the questionnaire scores. The main effects of emotion and fNMES were still significant, and the not-distracting subscale from the MAIA was also significant [β = −0.20, *z *= 2.13, 95% CI (−0.38, 0.02), SE = 0.10, *p* = 0.033]. This means that participants who were more in tune with unpleasant bodily sensations were more likely to categorise the face as happy, while participants who tended to ignore or distract from sensations of discomfort were less likely to do so. All other covariates were not significant (all *p* values > 0.130; Supplementary material B and C).

A second pre-registered model also included the fixed effect Ambiguity, where 30, 20 and 10% emotion intensity were coded as 0, 0.5 and 1 ambiguity. The model (conditional *R*^2^ = 0.60, marginal *R*^2^ = 0.56) revealed a main effect of Emotion [β = 1.09, *z *= 55.85, SE = 0.02, 95% CI (1.06, 1.14), *p* < 0.001] and an Ambiguity by Emotion interaction [β = −0.54, *z *= 18.58, SE = 0.03, 95% CI (−0.59, −0.48), *p* < 0.001]. All other main and interaction effects, including the fNMES by Ambiguity interaction of interest, were not statistically significant (all β values < 0.09, all *p* values > 0.240).

**Fig. 4.**
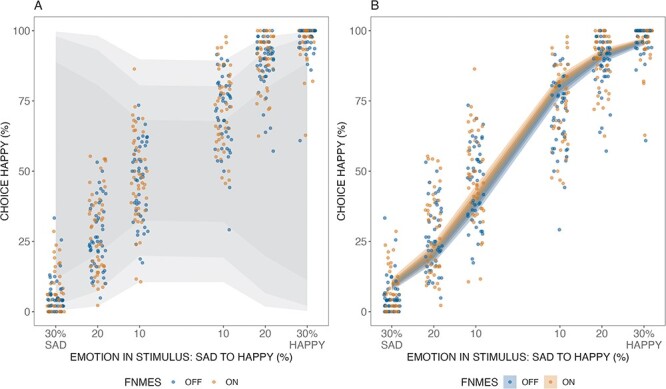
Prior and posterior predictions for the group-level effects of the Bayesian GLMM. Panel A represents the fixed effects, showing the percentage of choice happiness based on the percentage of emotion in the stimulus, ranging from sad to happy. The points are colour-coded by fNMES, and the shaded ribbon represents the uncertainty of the estimates. Panel B visualises the posterior predictions, with the ribbon showing uncertainty in the estimate, and points display individual participants (jittered for visibility).

Finally, we conducted a Bayesian GLMM analysis (not pre-registered), comparing two models. The full model on the response variable Choice (happy and sad) included the predictors fNMES, Emotion and their interaction (formula: Choice ∼ 0 + fNMES + fNMES:Emotion). A reduced model did not include fNMES (formula: Choice ∼ Emotion). Both models included a random intercept for the grouping effect of participants (formula: 0 + fNMES + fNMES:Emotion | participant) and were fitted using the brm function from the brm package (Bürkner, 2017). We used weakly informative priors assigned to the fNMES conditions, with a prior distribution of normal (0, 0.5) indicating that the effects were centred around zero with moderate uncertainty. To capture the interaction effect between fNMES and emotion, the coefficients were assigned normal priors of normal (0, 1). These priors indicate a belief that the interaction effects are centred around zero, with a higher uncertainty compared to the main effects (see Figure 4).

Model comparison was based on the computation of Leave-One-Out (LOO) and Widely Applicable Information Criterion (WAIC) weights. The LOO weights indicated that the full model weight was 0.72, while the model without the effect of fNMES had a weight of 0.28. This suggests that the full model is favoured by the data, as it has a higher weight than the alternative model. Similarly, the WAIC weights also favoured the full model, with a weight of 0.72, compared to 0.28 for the model without the effect of fNMES. Overall, our Bayesian analysis indicated that the full model, which includes the effect of fNMES, is preferred by the data.

### ERP analyses

**Fig. 5.**
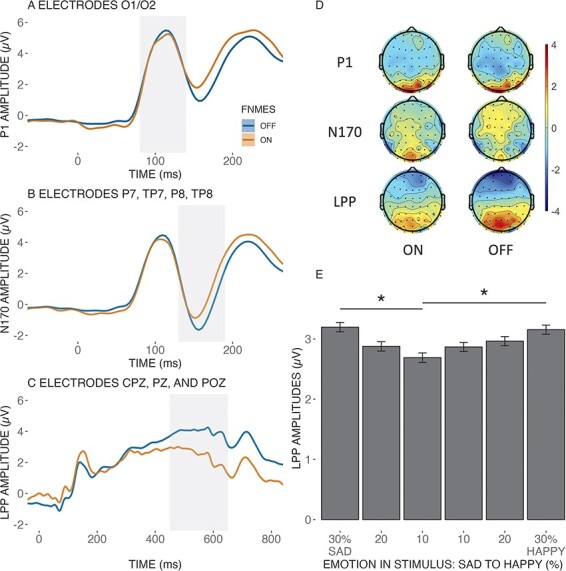
Panel A shows the time series of the ERP for the P1 component (80–140 ms), panel B shows the time series of the ERP for the N170 component (130–190 ms) and panel C shows the time series of the ERP for the LPP component (450–650 ms). The shaded grey area in each panel indicates the time region used for statistical analysis. Overall, after subtracting the fNMES-only trials, we observed a reduction in amplitude for all three components during fNMES on relative to fNMES off. Panel D shows the topography of each ERP component for its respective time, shaded in grey. Panel E shows the main effect of emotion on LPP amplitude, and error bars show the standard error. * < 0.05.

For the P1 component (conditional *R*^2^ = 0.92, marginal *R*^2^ = 0.01), a statistically significant main effect of fNMES was found [*F*(1, 407) = 7.36, *p* = 0.007], with larger amplitudes in the off (*M* = 4.23, SE = 0.46) compared to the on condition (*M* = 4.02, SE = 0.46). We did not observe significant effects of Emotion or fNMES by Emotion (all *F* values < 0.75 and all *p* values > 0.586). The second model (conditional *R*^2^ = 0.92, marginal *R*^2^ of 0.01) on the N170 amplitude revealed a main effect of fNMES [*F*(1, 407) = 31.17, *p* < 0.001], with larger negative amplitudes in the fNMES off (*M* = −2.10, SE = 0.46) compared to the on condition (*M* = −1.66, SE = 0.46). No other main or interaction effects were observed (all *F* values < 0.92 and all *p* values > 0.47). For the late positive potential (LPP) (conditional *R*^2^ = 0.76, marginal *R*^2^ of 0.13) a significant main effect of fNMES was found [*F*(1, 407) = 231.84, *p* < 0.001], with larger amplitudes in the off (*M* = 3.60, SE = 0.25) compared to the on condition (*M* = 2.29, SE = 0.25). A significant main effect of Emotion was also observed [*F*(5, 407) = 3.23, *p* = 0.007]. Bonferroni-corrected post hoc comparisons revealed larger amplitudes for faces displaying 30% compared to 10% sadness [*t*(407) = 3.430, *p* = 0.010, *M*_diff_ = 0.51; 95% CI (0.22, 0.80)] and for faces displaying 30% happiness compared to 10% sadness [*t*(407) = 3.05, *p* = 0.036, *M_diff_* = −0.45; 95% CI (−0.75–0.16)]. The interaction term was not statistically significant [*F*(5, 407) = 0.09, *p* = 0.993]. Figure 5 shows each ERP component’s time series, topographies and the main effect of emotion on the LPP component.

## Discussion

Whether the state of activation/relaxation of facial muscles can affect the visual perception and recognition of emotional faces remains debated (Hess and Fischer, 2014; Wood *et al*., 2016a). Advancing on that debate might require using methods that allow modulating facial feedback with great precision, such as computer-controlled fNMES (Efthimiou *et al*., 2023). Promising results of fNMES’ ability to modulate participants’ felt emotions and mood have been reported (Zariffa *et al*., 2014; Kapadia *et al*., 2019; Warren, 2021). It remains unknown, however, if the activation of specific facial muscles with fNMES can modulate the recognition of others’ facial expressions. We hypothesised that inducing a short and weak smile, by applying fNMES to the bilateral ZM muscle at the face onset and for 500 ms, would change face perception (measured with ERPs) and increase the likelihood of categorising emotionally ambiguous faces as happy.

Our main hypothesis was confirmed, as we demonstrated that by selectively engaging the ZM muscle through fNMES at stimulus onset, and for just 500 ms, we can change the way people perceive facial expressions—making them see emotionally ambiguous faces as happy (or at least as more happy than sad). This is a uniquely novel result, which aligns with prior research demonstrating the impact of facial muscle activity on facial emotion perception (Achaibou *et al*., 2008; Korb *et al*., 2014; Sel *et al*., 2015; Kuehne *et al*., 2021).

We found that the induction of a weak smile through fNMES leads to an increase of 2% (ß = 0.09) in the chance to categorise an ambiguous face as happy. This can be considered a small effect, which however stayed significant when statistically controlling for individual differences in positive or negative affective state, autistic traits, empathy, interoceptive awareness and discomfort. A small effect was moreover expected based on the literature (Coles *et al*., 2019), and statistical power was computed accordingly, as detailed in the pre-registration. Nevertheless, these findings should be considered as preliminary evidence, as this is the first study to investigate the influence of fNMES on the processing of emotional facial expressions, and Bayesian results were not entirely conclusive.

To investigate the potential influence of affective priming, participants’ affective states were assessed using the PANAS at both the start and end of the experiment. No statistically significant differences were observed between pre- and post-experiment PANAS scores, indicating no overall changes in affect after receiving fNMES to the ZM throughout many trials. Furthermore, recent research by Bulnes *et al*. (2023) sheds light on the drivers of facial feedback effects on face perception, specifically whether these effects emanate from motor matching or changes in affect. In their study, participants either mimicked the observed facial expressions of happiness and anger or imagined experiencing the emotions conveyed by the facial expressions. The findings revealed that face imitation resulted in immediate changes in emotion recognition, whereas the group that imagined feeling the observed expression performed similar to the control group. However, the group that imagined feeling the observed emotions exhibited improved performance on a subsequent emotion detection task administered at a later point in time. These findings suggest that both interventions exhibit task- and time-specific effects on emotion processing, with the imitation of observed facial expressions providing an immediate effect, while later effects are due to the imagining of the feelings. In our task, emotion categorisation was assessed immediately after facial muscle stimulation, indicating that the induced bias is, speculatively, driven by motor matching rather than changes in affect.

Results from the ERP data indicated significantly smaller P1, N170 and LPP components during fNMES. A speculative explanation for this finding is that the fNMES-induced activation of smiling muscles shifted the relative weights of visual and proprioceptive processing: turning up the signal coming from facial muscles could reduce the visual system’s workload in discerning facial details (Achaibou *et al*., 2008). Thus, the reduction in amplitude across all visual components may be attributed to the central nervous system prioritising the processing of proprioceptive signals induced by fNMES to the ZM. That is, visual processing is dampened given an alternative sensory input that might aid in resolving the ambiguous facial expression. This is also in line with recent work showing that mu desynchronisation to emotional faces—considered to reflect the engagement of the mirror neuron system—is reduced when participants hold a pen in their mouth (Birch-Hurst *et al*., 2022).

We also found a main effect of emotion on the LPP, with larger amplitudes when the faces were less ambiguous. This finding aligns well with previous research showing that this period is when the resolution of emotional ambiguity occurs (Calvo *et al*., 2013). No main effect of emotion was found for the P1 and N170 components, which is likely due to our stimulus set consisting of highly ambiguous facial expressions, which the early components are less sensitive to (Eimer, 2011; Hietanen and Astikainen, 2013). Moreover, the literature is mixed on whether P1 and N170 amplitudes are modulated by emotional expressions or not (Achaibou *et al*., 2008; Sel *et al*., 2014).

Notably, our facial manipulation technique did not interact with the emotional content of the facial expressions, in contrast to previous studies utilising the pen-in-mouth technique that showed increased N170 amplitudes to facial expressions during smile production (Sel *et al*., 2015; Kuehne *et al*., 2021). Several factors may account for this discrepancy. Firstly, our sample size was determined based on a power analysis for the main effect of fNMES in the categorisation choices, potentially leading to an underpowered EEG analysis and the inability to detect an interaction between fNMES and the emotional content of facial expressions. Secondly, our facial manipulation differed from previous studies, which utilised the pen-in-mouth technique, whereas our study was the first to incorporate fNMES alongside EEG. Consequently, the functional implications of the observed fNMES main effects on ERPs remain unclear. To gain further insights into this phenomenon, future research should investigate the effects of stimulating different facial muscles, such as the ‘depressor anguli oris’ or ‘corrugator’ muscles, on visual processing.

This research contributes to the emerging field of fNMES applications in manipulating facial expressions and sheds light on the potential influence of subtle facial muscle stimulation on visual perception. Our behavioural findings have positive implications for facial feedback interventions that utilise posing as a method to reduce or manage distress (Ansfield, 2007) and address symptoms of depression (Finzi and Rosenthal, 2014, 2016; Fromage, 2018). Over-the-counter electrical stimulation devices, commonly used for pain management in the face, body, and craniofacial disorders (Efthimiou *et al*. 2023), may be harnessed to assist individuals with conditions that impede facial feedback, such as Bell’s palsy (Alakram and Puckree, 2010), Moebius syndrome (Stefani *et al*., 2019) and Parkinson’s disease (Argaud *et al*., 2018). Furthermore, this technique holds promise for future research that can investigate the time course of facial feedback effects, specifically examining whether stimulus-congruent facial feedback occurs only after early visual processing has been completed (Argaud *et al*., 2018; Niedenthal, 2007; Halberstadt *et al*., 2009).

The present study has several limitations that should be acknowledged. First, we only targeted the ZM muscle (AU12) to induce a weak smile, while a prototypical expression of happiness is stronger and often involves the activation of the OO muscle (AU6). This limited focus may partially explain the weak effect of fNMES on categorisation choices observed in our study. Future research should explore the impact of targeting both AU12 and AU6 to induce a more robust effect of fNMES on emotional perception (although small changes in AU6 activity were observed during fNMES targeting the ZM, see Figure 2).

Second, our study was limited to investigating the effect of fNMES on positive facial expressions (smiling), while the impact of fNMES on negative expressions (frowning) remains unknown. Future research should explore whether fNMES can modulate the processing of negative emotions and how this effect may differ from that observed for positive emotions. Similarly, the absence of a non-face control condition limits our ability to definitively determine whether the observed effects are primarily driven by changes in felt emotion or motor matching. Future research incorporating a non-face control condition, such as neutral objects or non-facial stimuli, could help clarify this distinction especially as they do not induce spontaneous mimicry. If the effects are primarily attributed to changes in felt emotion, then these effects should be observed even in response to non-face stimuli, as the motor feedback from facial expressions would still be present. However, if the effects are primarily driven by motor-matching mechanisms, then they would be specific to facial stimuli, as the motor feedback would only be relevant in the context of faces.

Third, it cannot be entirely ruled out, at this stage, that the main fNMES effect on the P1, N170 and LPP components is not at least partly due to our data treatment. Indeed, to remove somatosensory evoked potentials affecting central electrodes but possibly also occipitotemporal areas, brain activity during fNMES-only trials was subtracted from that during the trials including both fNMES and face presentation. The same correction was not applied to fNMES off trials, which did not contain somatosensory evoked activity. A potential way to overcome this problem is to compute difference scores between emotion levels (30% *vs* 10%) and compare them across fNMES conditions. This approach did not result in differences between fNMES conditions (see Supplementary material D), possibly due to the use of weak emotional expressions.

Finally, our study only administered fNMES at face onset and did not investigate the impact of altering the timing of fNMES delivery relative to the stimulus, which can be seen as a limitation. We focused on a single time point for fNMES stimulation to enhance statistical power and provide a first proof-of-concept for fNMES influence on visual perception. Future research should, however, attempt to investigate the effects of fNMES delivery at different time points during facial processing. For example, Pitcher *et al*. (2008) investigated the role of the visual and somatosensory cortexes in facial emotion discrimination by delivering transcranial magnetic stimulation (TMS) in seven time windows covering the time from 20 to 290 ms after the onset of an emotional face. The authors showed that emotion discrimination accuracy was only reduced after early (60–100 ms after face onset) inhibition of the right occipital face area and somewhat later (100–170 ms) inhibition of the right somatosensory cortex. Our fNMES delivery period covered (and exceeded) these ranges but does not inform us when fNMES should best be delivered. It might even be that the effects of proprioceptive facial feedback on facial emotion recognition are more pronounced when fNMES is delivered later than the time window targeted here. Indeed, when shown emotional faces participants typically react by imitating them, but in the EMG signal the earliest occurrence of facial mimicry is around 200–300 ms after stimulus onset (Achaibou *et al*., 2008; Korb *et al*., 2014) and a frequent finding is that facial mimicry becomes statistically significant even later than that, from 500 ms onwards (Dimberg, 1988). Therefore, delivering fNMES at 500 ms might prove even more effective, and in any case, could provide further insights into the temporal dynamics of facial feedback’s influence on emotion recognition.

In conclusion, our study provides the first demonstration that providing controlled weak electrical stimulation to specific facial muscles, at a precise time and for a precise short duration of 500 ms, can shape how ambiguous facial expressions are perceived. This finding supports the notion that facial muscle activity and the processing of affect are inherently linked and have potential implications for the treatment of affective disorders and the study of embodied cognitive processing.

## Supplementary Material

nsae013_Supp

## Data Availability

The data and materials for all experiments are available at osf.io/vbnyx.
