# Supplementary material for: Zygomaticus activation through facial neuromuscular electrical stimulation (fNMES) induces happiness perception in ambiguous facial expressions and affects neural correlates of face processing
Source: Soc Cogn Affect Neurosci. 2024 Feb 8;19(1):nsae013. doi: 10.1093/scan/nsae013 (PMC10873823; doi:10.1093/scan/nsae013)
Supplement: nsae013_Supp [file nsae013_supp.zip › scan-23-254-File002.docx]

Supplementary material

Supplementary material A.

Output from the lmerTest model for the pre-registered model which included an interaction between the predictor's emotion and fNMES in the random effects structure. This model resulted in a singular fit.

Table 1

Model summary for the pre-registered analysis, which included a full random effects structure.

|  | **Choice** | | |
| --- | --- | --- | --- |
| Predictors | β | CI | *p* |
| (Intercept) | 0.49 | 1.36 – 1.96 | **<.001** |
| Emotion | 0.92 | 2.37 – 2.67 | **<.001** |
| fNMES | 0.10 | 1.02 – 1.19 | **.020** |
| Emotion * fNMES | 0.91 | 0.97 – 1.06 | .660 |
| **Random Effects** | | | |
| σ^2^ | 3.29 | | |
| τ_00_ _participant_ | 0.37 | | |
| τ_11_ _participant.Emotion_ | 0.03 | | |
| τ_11_ _participant.fNMES_ | 0.01 | | |
| τ_11_ _participant.Emotion:fNMES_ | 0.00 | | |
| ρ_01_ | 0.45 | | |
|  | 0.76 | | |
|  | 0.01 | | |
| N _participant_ | 47 | | |
| Observations | 26811 | | |
| Marginal R^2^ / Conditional R^2^ | .55 / NA | | |

Supplementary material B.

Summary of a Generalised Linear Mixed Model (GLMM) predicting participants' choice with covariates. We fitted a GLMM (*n* = 45) to predict Choice (happy or sad) with the predictor variables in the table below. Formula: Choice ~ Emotion * fNMES + ASQ + EQ +

MAIA Noticing + MAIA not-worrying + MAIA Attention regulation + MAIA Emotional Awareness + MAIA not-Distracting + MAIA Self-regulation + MAIA Body Listening + MAIA Body trusting + (Emotion + fNMES | participant).

Table 2

Summary of the model including the MAIA, ASQ, and EQ questionnaire. Shaded rows show statistically significant results.

| Predictor | β | *SE* | z | *p* |
| --- | --- | --- | --- | --- |
| Emotion | 1.96 | .07 | 28.23 | < .001 |
| fNMES | .09 | .04 | 2.41 | .016 |
| ASQ | -.15 | .10 | -1.51 | .130 |
| EQ | .02 | .10 | .16 | .875 |
| MAIA: Noticing | -.02 | .10 | -.23 | .817 |
| MAIA: Not-Worrying | -.04 | .10 | -.39 | .698 |
| MAIA: Attention regulation | -.05 | .12 | -.44 | .661 |
| MAIA: Emotional Awareness | .04 | .11 | .34 | .732 |
| MAIA: Not-Distracting | -.19 | .09 | -2.13 | .033 |
| MAIA: Self-Regulation | .08 | .12 | .71 | .478 |
| MAIA: Body Listening | -.05 | .13 | -.43 | .670 |
| MAIA: Body trusting | .10 | .10 | 1.02 | .390 |
| Emotion * fNMES | .01 | .04 | .16 | .874 |

Supplementary material C.

Summary of the Generalised Linear Mixed Model (GLMM) predicting participants' choice with covariates. We fitted a GLMM (*n* = 41) to predict Choice (happy or sad) with the predictor variables in the table below. Formula: Choice ~ Emotion * fNMES + Positive Affect + Negative Affect + Discomfort + (Emotion + fNMES | participant).

Table 3

Summary of the model including the covariates PANAS and discomfort. Shaded rows show statistically significant results.

| Predictor | β | *SE* | z | *p* |
| --- | --- | --- | --- | --- |
| Emotion | 2.00 | 0.07 | 27.27 | < .001 |
| fNMES | -0.01 | 0.02 | 2.38 | .017 |
| Positive Affect Time 1 | 0.12 | 0.09 | 1.27 | .204 |
| Negative Affect Time 1 | -.01 | 0.09 | -0.13 | .894 |
| Discomfort | -0.07 | 0.10 | -0.67 | .502 |
| Emotion * fNMES | -0.02 | 0.02 | -0.77 | .443 |

Supplementary material D.

To make sure the fNMES-only subtraction method did not contribute to the main fNMES effects found on each ERP component, we calculated four emotion difference scores, controlling for fNMES. Specifically, we subtracted the average activity in response to 10% happy and sad faces (the most ambiguous emotion levels) from the activity elicited by 30% happy/sad faces (the least ambiguous emotion levels). We did this for each component (P1, N170, and LPP) and for each fNMES condition (e.g., fNMES on and fNMES off; for the equation see below). We then compared these difference scores for the fNMES on and off conditions.

Happy On = fNMES on (30% happy – (average of 10% Happy and 10% Sad)

Happy Off = fNMES off (30% happy – (average of 10% Happy and 10% Sad)

Sad On = fNMES on (30% happy – (average of 10% Happy and 10% Sad)

Sad Off = fNMES off (30% happy – (average of 10% Happy and 10% Sad)

Table 4

Paired sample t-tests comparing fNMES off to on, for each ERP component, after computing difference scores between emotion levels.

| Component | Emotion | *t* | *CIs* | *M_diff_* | *p* |
| --- | --- | --- | --- | --- | --- |
| P1 | Happy | -0.39 | -0.46, 0.32 | -0.08 | .699 |
|  | Sad | -0.16 | -0.47, 0.40 | -0.04 | .871 |
| N170 | Happy | -1.17 | -0.61, 0.04 | -2.85 | .084 |
|  | Sad | -0.04 | -0.34, 0.33 | -0.01 | .965 |
| LPP | Happy | -0.29 | 0.36, 0.27 | -0.05 | .769 |
|  | Sad | 0.30 | -0.23, 0.31 | 0.04 | .767 |

Note. All t-tests have a *df* of 37.
